# 9.2 BRAIN STRUCTURAL AND NEUROCHEMICAL HETEROGENEITY AND HOMOGENEITY IN PSYCHOTIC DISORDERS: TRANSDIAGNOSTIC PET AND MRI IMAGING FINDINGS IN SCHIZOPHRENIA AND BIPOLAR AFFECTIVE DISORDER

**DOI:** 10.1093/schbul/sby014.030

**Published:** 2018-04-01

**Authors:** Oliver Howes, Sameer Jauhar, Stefan Brugger, Fiona Pepper

**Affiliations:** 1 MRC LMS and KCL; 2 King’s College London & MRC Clinical Sciences Centre; 3 MRC LMS

## Abstract

**Background:**

Psychosis is seen in a number of disorders and treated with the same drugs. However, there is considerable variability in response to treatment and clinical course. Understanding the neurobiology underlying psychosis across diagnoses and in treatment response is important to help guide the development of new treatments and biomarkers for treatment response. Elevated dopamine synthesis and release capacity and structural brain changes have been consistently associated with schizophrenia, but it remains unknown how variable these are, or how they compare across psychotic disorders.

**Methods:**

Two cohorts of first episode patients, one with a diagnosis of schizophrenia (n=16) and another with a diagnosis of bipolar affective disorder (n=22) received 18F-DOPA PET and [1H]-MR spectroscopy imaging and clinical measures. All patients had experienced a psychotic episode and received clinical follow-up over 18 months to determine diagnostic stability. We then conducted a meta-analysis using a novel meta-analytic approach to quantify variability in measures to investigate structural and neurochemical heterogeneity in schizophrenia and bipolar affective disorder. The entire PubMed, EMBase and PsychInfo databases were searched from inception to identify relevant studies and the natural log of the measures of dispersion and the coefficient of variance.

**Results:**

Striatal dopamine synthesis capacity (Kicer) was significantly elevated in both bipolar (effect size=1.02; p<0.003) and schizophrenia (effect size=0.9; p<0.05) groups, compared to controls. There was no significant difference in dopamine synthesis capacity between bipolar and schizophrenia groups (p>0.4). Kicer was significantly positively correlated with positive psychotic symptom severity in the transdiagnostic group of people with psychosis (r=0.52, p<0.004), and in the bipolar group after adjusting for manic symptom severity (r=0.6, p<0.01). There were no differences in glutamate levels in the anterior cingulate cortex.

In the meta-analyses a total of 128 studies were identified including >4000 patients and >4000 controls. Variability ratio was significantly increased in patients relative to controls in gray matter volumes in temporal lobe (VR=1.1, p=0.004) and thalamus (VR=1.16, p<0.001), and in striatal dopamine receptor density (p<0.05) but unaltered in frontal cortex and significantly reduced in the anterior cingulate cortex (VR=0.9, p=0.02)

**Discussion:**

Elevated dopamine synthesis capacity is associated with psychosis across diagnostic boundaries and linked to the severity of psychotic symptoms, even after adjusting for manic symptom severity. Striatal dopamine receptor density and structural gray matter volumes in a number of cortical and sub-cortical regions show heterogeneity in psychotic disorders, but frontal cortical regions show unaltered and, in the case of the anterior cingulate cortex, reduced heterogeneity, suggesting alterations are homogenous across patients. Taken together these findings striatal dopamine synthesis and structural changes in frontal cortex are common mechanisms linked to psychosis across disorders.

